# Dual Inhibition of mTORC1/2 Reduces Migration of Cholangiocarcinoma Cells by Regulation of Matrixmetalloproteinases

**DOI:** 10.3389/fcell.2021.785979

**Published:** 2022-01-13

**Authors:** Katharina Joechle, Huda Jumaa, Kerstin Thriene, Claus Hellerbrand, Birte Kulemann, Stefan Fichtner-Feigl, Sven A. Lang, Jessica Guenzle

**Affiliations:** ^1^ Department of Surgery and Transplantation, University Hospital RWTH Aachen, Aachen, Germany; ^2^ Department of General and Visceral Surgery, Medical Center—University of Freiburg, Faculty of Medicine, Freiburg, Germany; ^3^ Faculty of Medicine, Medical Center—University of Freiburg, Freiburg, Germany; ^4^ Institute of Biochemistry, Friedrich-Alexander University Erlangen-Nürnberg, Erlangen, Germany; ^5^ Department of Surgery, University Medical Center Schleswig-Holstein Campus Lübeck, Lübeck, Germany; ^6^ Comprehensive Cancer Center Freiburg-CCCF, Medical Center-University, Freiburg, Germany

**Keywords:** MTORC1/2, cholangiocarcinoma, migration, invasion, matrix metalloproteinases

## Abstract

Cholangiocarcinoma (CCA) is a rare but highly aggressive tumor entity for which systemic therapies only showed limited efficacy so far. As OSI-027—a dual kinase inhibitor targeting both mTOR complexes, mTORC1 and mTORC2 - showed improved anti-cancer effects, we sought to evaluate its impact on the migratory and metastatic capacity of CCA cells *in vitro.* We found that treatment with OSI-027 leads to reduced cell mobility and migration as well as a reduced surviving fraction in colony-forming ability. While neither cell viability nor proliferation rate was affected, OSI-027 decreased the expression of MMP2 and MMP9. Moreover, survival as well as anti-apoptotic signaling was impaired upon the use of OSI-027 as determined by AKT and MAPK blotting. Dual targeting of mTORC1/2 might therefore be a viable option for anti-neoplastic therapy in CCA.

## Introduction

Cholangiocarcinoma (CCA) arising from epithelial cells of the biliary tract, can be subdivided into an intra- and extrahepatic subtype (iCCA and eCCA) depending on the anatomic localization. While CCA is currently considered a rare cancer entity, its incidence is constantly increasing worldwide. Radical surgery remains the only curative option but recurrence is common even after complete tumor removal. In case of irresectable disease, systemic therapy based on gemcitabine and cisplatin is applied with limited success. Even more, no targeted therapy has entered the clinical routine for advanced CCA although several efforts have been made to unravel the molecular basis of the disease (Brindley et al., 2021). Hence, the prognosis of CCA remains poor and novel insights and strategies are urgently needed for targeting CCA.

Several studies indicate that activation and dysregulation of the serine/threonine kinase mammalian target of rapamycin (mTOR) are associated with tumorigenesis but also processes like tumor growth, metastasis, and drug resistance in various tumor entities (Popova and Jücker 2021). In general, this makes mTOR an interesting target for anti-neoplastic therapy. However, mTOR resides in two structurally distinct multiprotein complexes, mTORC1 and mTORC2, distinguished by different co-factors, with RAPTOR and RICTOR being specific for either complex (Saxton and Sabatini 2017). While mTORC1 is extensively studied mainly due to the availability of pharmacological mTORC1 inhibitors, the role of mTORC2 in cancer is less clear. Inhibitors such as rapamycin and everolimus, also known as rapalogs, primarily target mTORC1 by allosteric inhibition but direct inhibition of mTORC2 is lacking and, even more, mTORC2 effectors (e.g., AKT) can be activated via feedback mechanisms upon treatment with rapalogs (O’Reilly et al., 2006; Lang et al., 2010). However, effective pan-mTOR inhibition is eligible as mTORC2 is supposed to be a promising target for anti-cancer therapy.

Direct targeting of RICTOR, the crucial component of mTORC2, led to impairment of tumor growth *in vitro* for CCA. Moreover, cholangiocarcinogenesis was completely abrogated upon liver-specific RICTOR knockout in mice (Zhang et al., 2017). In addition, enhanced apoptosis and reduced cell proliferation were detected upon si-RNA mediated RICTOR knockdown and simultaneous treatment with sorafenib in CCA (Ding et al., 2016). While these data show the importance of mTORC2 inhibition in CCA for anti-cancer treatment effects might even be potentiated when combining mTORC2 with mTORC1 inhibition as everolimus is also known to lead to decreased cell proliferation in CCA *in vitro* (Rattanasinganchan et al., 2006)*.* A new generation of mTOR inhibitors has been developed for targeting both, mTORC1 and mTORC2, with the aim to overcome the limitations of sole mTORC1 inhibition by blocking the ATP-binding pocket of the complexes at the active site (Schenone et al., 2011). These so-called dual kinase inhibitors such as OSI-027 show a strong suppression of both mTORC1- and mTORC2-mediated downstream signaling in various tumor cell lines, which translates into an improved anti-neoplastic activity *in vitro* and *in vivo* (Bhagwat et al., 2011)*.* Hence, dual kinase inhibition might be a promising new therapy for CCA. Reduced cell proliferation *in vitro* and partial tumor regression *in vivo* were achieved with MLN0128 and AZD8055 treatment (Ewald et al., 2014; Zhang et al., 2017). However there are no data on the effects of dual kinase inhibition on metastasis and tumor cell migration for CCA so far and clinical trials are warranted to prove the efficacy in patients (Bendell et al., 2015; Mateo et al., 2016; Statz et al., 2017). In summary, targeting mTORC2 seems to be an interesting option for anti-cancer treatment in CCA.

The ability to degrade extracellular proteins is crucial for regular cell migration as well as for metastasis of tumor cells. Metastasis is a complex interaction between tumor cells and the extracellular matrix. Various proteolytic enzymes are involved in the degradation of the tumor environment such as the matrix metalloproteinases (MMPs). Under physiological conditions, MMPs are involved in inflammation processes, proliferation and differentiation, and are therefore latently expressed. In solid tumors, the expression level of many MMPs can be correlated with the malignancy and invasive capacity as well as a poor prognosis. Interestingly, regulation of MMPs at least in part via the mTOR pathway has been described (Liang et al., 2017). Furthermore, we have recently described that targeting mTORC2 with a novel specific inhibitor modulates MMP expression in melanoma cells (Guenzle et al., 2020). Therefore, MMP expression might also be susceptible for targeting mTORC1 and mTORC2.

The main objective of the present study was to investigate the effect of the dual kinase inhibitor OSI-027 on the migration and invasion capacity of cholangiocarcinoma cells *in vitro*. We provide evidence that dual kinase inhibition of mTORC1/2 regulates survival and proliferation by impaired AKT and MAPK signaling as well as migration processes by decreased expression of MMP2 and MMP9 in cholangiocarcinoma.

## Materials and Methods

### Cell Culture

The human intrahepatic cholangiocellular carcinoma (iCCA) cell lines HuCCA1 and HuCCT1 (JCRB cell bank) cultured in DMEM containing 10% fetal bovine serum at 37 °C in a humidified atmosphere containing 5% CO_2_. The dual mTORC1/2-inhibitor OSI-027 was obtained from Selleckchem, United States, and dissolved in DMSO. Cells were exposed to OSI-027 concentrations between 100 and 1500 nM for up to 72 h. The final concentration of DMSO was not higher than 0.01% (when using 1500 nM OSI-027).

### Migration Assay

Cells were seeded in 24 well plates with silicon bars to create a gap of about 2 mm as described before and treated with OSI-027 up to 500 nM for 120 h (Guenzle et al., 2017). The time required for wound closure was measured at three independent points in each image by microscopy (Leica DMIL LED, Camera Leica DFC450C) and documented up to 120 h.

### Clonogenic Assay

Cells were seeded in flasks and exposed to up to 500 nM OSI-027 for 48 h. 500 cells were reseeded in 6-well plates and cultured in complete DMEM. After 2 weeks, cells were washed with PBS, fixed in PFA for 30 min, and stained with hematoxylin for 30 min. Plates were washed with water and air-dried. Counting of colonies was performed by microscopy (Leica DMIL LED, Camera Leica DFC450C) and differed in the colony types holoclone, meroclone, and paraclone (Barrandon and Green 1987; Beaver et al., 2014; Guenzle et al., 2020). Plating efficiency (PE) and Surviving fraction (SF) was calculated and set to control to 100% using following equation: PE = # of colonies formed/# of cells seeded x 100%; SF = # colonies formed after treatment/(# of cells seeded x PE).

### Viability Assay

Cell viability was determined by MTT Assay. 5 × 10^4^ cells were grown in 96 well plates with complete DMEM and treated with 100–1500 nM OSI-027 for up to 72 h. MTT Assay was performed as described before (Guenzle et al., 2019). Absorbance at 570 nm was measured (Tecan, Männedorf, Switzerland). Percentages were calculated relative to the viability of untreated controls set to 100%. The experiment was done in triplicate and is representative of a minimum of three independent studies.

### Cell Proliferation Assay

Cell proliferation was monitored by 5′-bromodeoxyuridine (BrdU) incorporation assay (Roche Diagnostics, Mannheim, Germany). 5 × 10^4^ cells were seeded in 96 well plates and treated with OSI-027 up to 1500 nM. Cells were stained with BrdU following the manufacturer’s instructions. The percentage of cells exhibiting genomic BrdU incorporation was measured by absorbance at 370 nm (Tecan, Männedorf, Switzerland). Percentages were calculated relative to the proliferation of untreated controls set to 100%.

### Cell Cycle Analysis

Cell cycle phases were analyzed by flow cytometry. Treated cells were fixed with 4% PFA for 20 min at RT. Permeabilization was performed with 0.05% triton X-100 for 15 min before staining with DAPI for 20 min at RT. 10 000 cells were acquired by flow cytometry at FACSCalibur (Beckman Coulter, Brea, CA, United States). The populations of cells in Go-G1 phase, S phase and G2-M phase were analyzed by FlowJo software (BD group, Ashland, OR, United States).

### Western Blotting

Cholangiocellular carcinoma cells were cultured in serum-free DMEM. Supernatant of OSI-027 treated cells was collected. Equal amounts of protein (4 µg of supernatant, 20 µg of lysate) were applied on 10% SDS-polyacrylamide gels and electrophoresed (BioRad, Munich, Germany) as described before (Guenzle et al., 2017). Blots were incubated with primary antibodies anti-MMP2 (#4022 1:1000 Cell Signaling Technology), anti-AKT (#9272 1:1000 Cell Signaling Technology), anti-pAKT^Ser473^ (#9271 1:1000 Cell Signaling Technology), anti-ERK1/2 (#4696 1:1000 Cell Signaling Technology), anti-pERK^Thr202/Tyr204^ 1/2 (#4370 1:1000 Cell Signaling Technology), anti-p38 (#8690 1:1000 Cell Signaling Technology), anti-p-p38^Thr180/Tyr182^ (#4511 1:1000 Cell Signaling Technology), anti-4EBP1^Ser65^ (#9451 1:1000 Cell Signaling Technology), anti-p-p70S6K^Thr389^ (#9206 1:1000 Cell Signaling Technology), anti-pSTAT3^Tyr705^ (#9145 1:1000 Cell Signaling Technology), anti-PDI (sc-74551 1:1000 Santa Cruz). Proteins were visualized by enhanced chemiluminescence (BioRad, Munich, Germany). PDI was used as loading control for the whole cell lysate. Finally, densitometry of the presented western blot was performed using ImageJ. Ratio was calculated to pixel/area of reference PDI. Expression of phosphorylated protein was calculated in relation to total protein amount.

### Gelatin Zymography

Gelatin is a substrate of MMP2 and can be used for detection of activity in supernatant (Guenzle et al., 2017). Equal amounts of supernatant (4 µg) used for western blot were applied under non-reducing conditions on 10% copolymerized gelatin-polyacrylamide gels and electrophoresed (BioRad, Munich, Germany) in running buffer (25 mM Tris, 192 mM glycine, 0.1% SDS). Gels were washed twice in 2.5% Triton-X-100/ddH_2_O for 15 min following incubation in development buffer (50 mM Tris, 5 mM CaCl_2_, 0.02% NaN_3_) for 4 h. Gels were fixed by shaking in methanol:ethanol:acetic-acid (4.5:4.5:1) for 15 min and stained with fixation buffer containing 0.1% Coomassie for 2 h. Gels were incubated in fixation buffer until transparent bands appeared. Gels were visualized by ChemiDoc (BioRad, Munich, Germany).

### Statistical Analysis

All experiments were performed in triplicates. Data are shown as mean ± SD and were compared using an unpaired two-tailed student’s t-test; *p* < 0.05 was considered statistically significant. Asterisks (* *p* ≤ 0.05, ** *p* ≤ 0.01, *** *p* ≤ 0.001) indicate significance.

## Results

### Dual Inhibition of mTORC1/2 Reduces Migration Capacity in a Time- and Dose-dependent Manner

**FIGURE 1:**
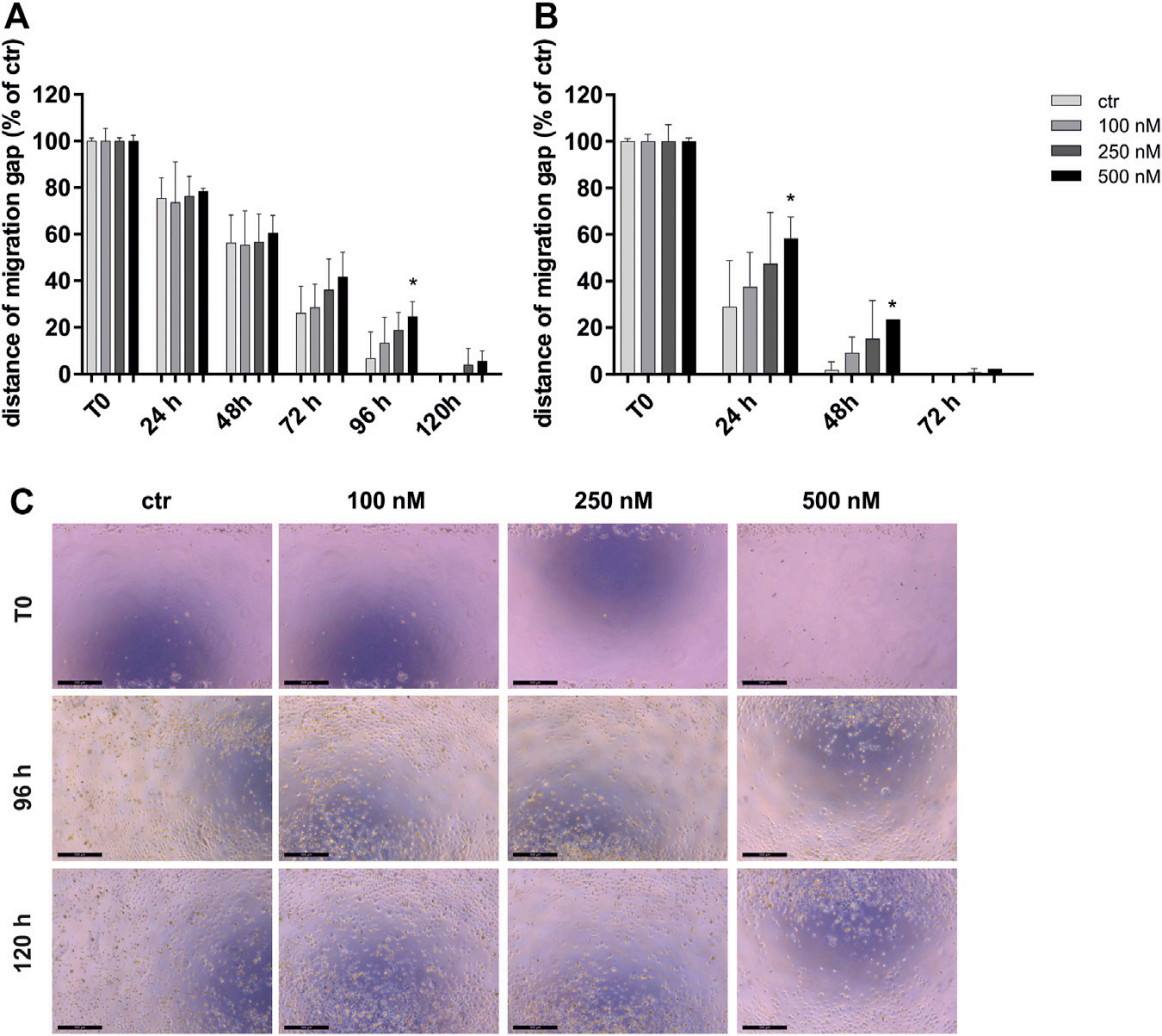
MTOR1/2 blockade inhibits migration capacity dose-dependently. Migration capacity of HuCCA1 **(A)** and HuCCT1 **(B)** was investigated after exposure of 100–500 nM OSI-027 by wound closure assay over 120 h and displayed as distance of the migration gap. Treated cells were normalized to T0 (start of the experiment) set to 100% (±SD of three independent experiments). Asterisks (* *p* ≤ 0.05) indicate significance between treatment and the control. Representative images **(C)** show the initial gap (T0) and the gap after 96 h as well as 120 h of control and treatment of HUCCA1 with 100–500 nM OSI-027. The initial width of the migration gap was 2 mm and is shown as the distance of the migration gap. Scale bar indicates 500 µm.

To investigate the effect of OSI-027 on the migration capacity of CCA cells, we performed a wound closure assay over 120 h for HuCCA1 and HuCCT1 cells exposed to 100–500 nM of the dual kinase inhibitor (Figure 1). Control cells, not exposed to OSI-027 closed the gap within 120 h (HuCCA1) or 72 h (HuCCT1). Starting at 24 h, the ability to close the migration gap is time- and dose-dependently reduced in HuCCT1 (Figure 1B) more than in HuCCA1 (Figure 1A). While HuCCA1 cells almost closed the initial gap within 96 h, cells exposed to 500 nM OSI-027 still exhibited a gap of about 25% (*p* = 0.03) as demonstrated in Figure 1C. However, HuCCT1 cells closed the gap within 48 h, cells exposed to 500 nM OSI-027 still exhibited a gap of about 24% (24 h, *p* = 0.04; 48 h, *p* = 0.03).

**FIGURE 2:**
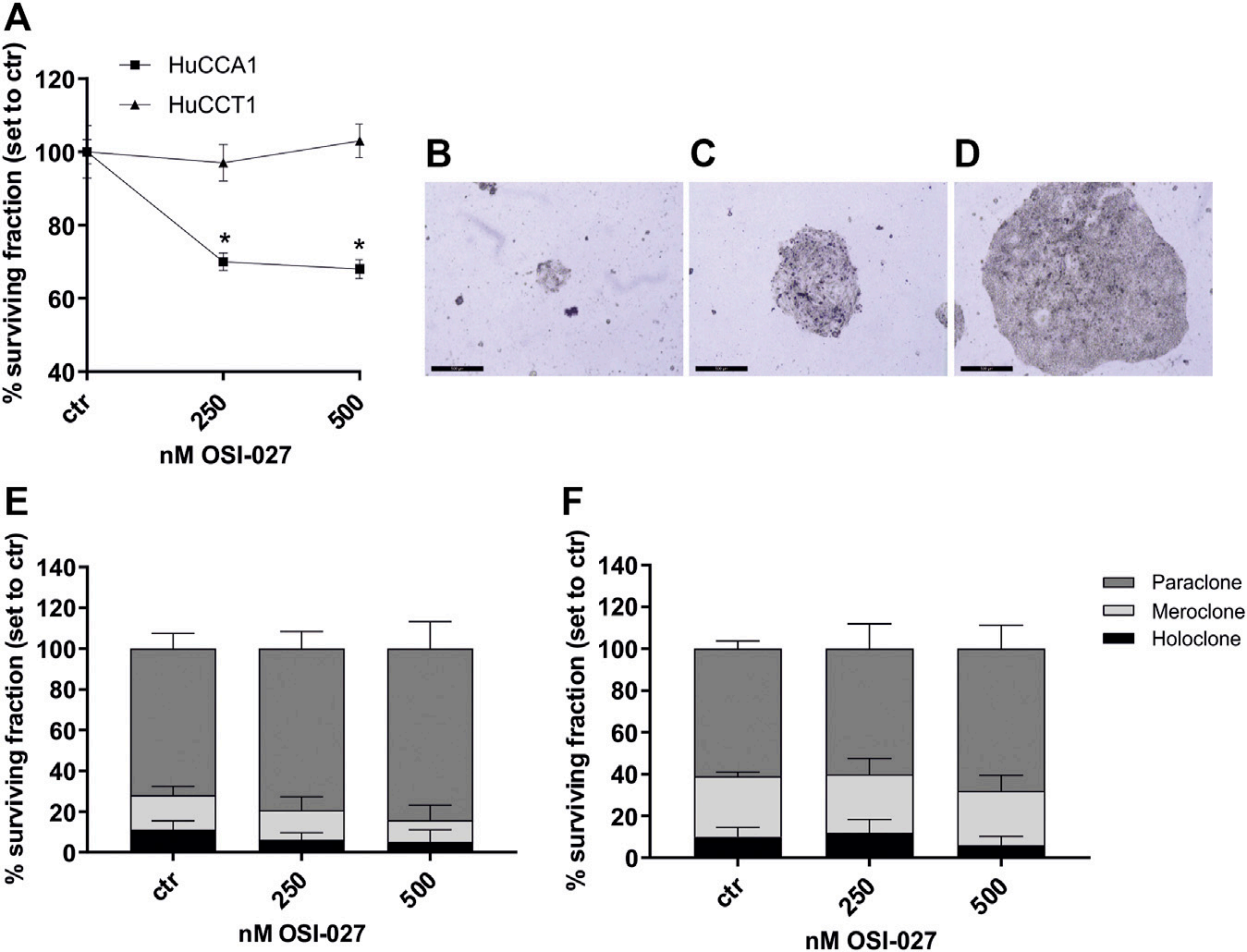
Dual inhibition of mTORC1 and mTORC2 reduces surviving fraction but not colony-forming ability. Treatment with OSI-027 (250 and 500 nM) for 48 h decreased the surviving fraction significantly in CCA cells HuCCA1 over 2 weeks (*p* < 0.03) **(A)**. Representative images **(B–D)** show the characteristic morphology of the para-, mero- and holoclones of HuCCT1. The morphology of the colonies and the colony-forming ability of HuCCA1 **(E)** and HuCCT1 **(F)** did not alter significantly in the distribution of para-, mero- and holoclones. Asterisks (* *p* ≤ 0.05) indicate significance between treatment and control. Experiments were performed in triplicates. Scale bar indicates 500 µm.

The cell surviving fraction (SF) was calculated based on a clonogenic assay over 2 weeks and is dependent on the dose of 250 and 500 nM of the dual mTORC1/2 inhibitor OSI-027. After exposure of OSI-027, HuCCA1 revealed a significantly reduced SF of 68% compared to control (*p* = 0.03) (Figure 2A). As described by Barrandon and Green, colonies of CCA cells were subdivided into holoclone-, paraclone- and meroclone-like colonies based on their morphology as demonstrated in Figures 2B–D. Subdivided fractions of non-treated HuCCA1 cells revealed 11% holoclone-like cells exhibiting differentiated cells with high growth potential in cell-rich colonies, 17% meroclone-like, and 72% paraclone-like colonies (Figure 2E). HuCCT1 cells exhibited 10% of holoclone-like cells, 29% of meroclone-like, and 61% of paraclone-like colonies (Figure 2F). Treatment with 250 nM or 500 nM OSI-027 did not alter this ratio in a significant manner. Taken together, dual kinase inhibition of mTORC1/2 leads to reduced cell mobility and migration as well as a reduced surviving fraction in colony-forming ability in CCA cells.

### Dual Inhibition of mTORC1/2 Does Not Affect Cell Viability and Proliferation of CCA Cell Lines

**FIGURE 3:**
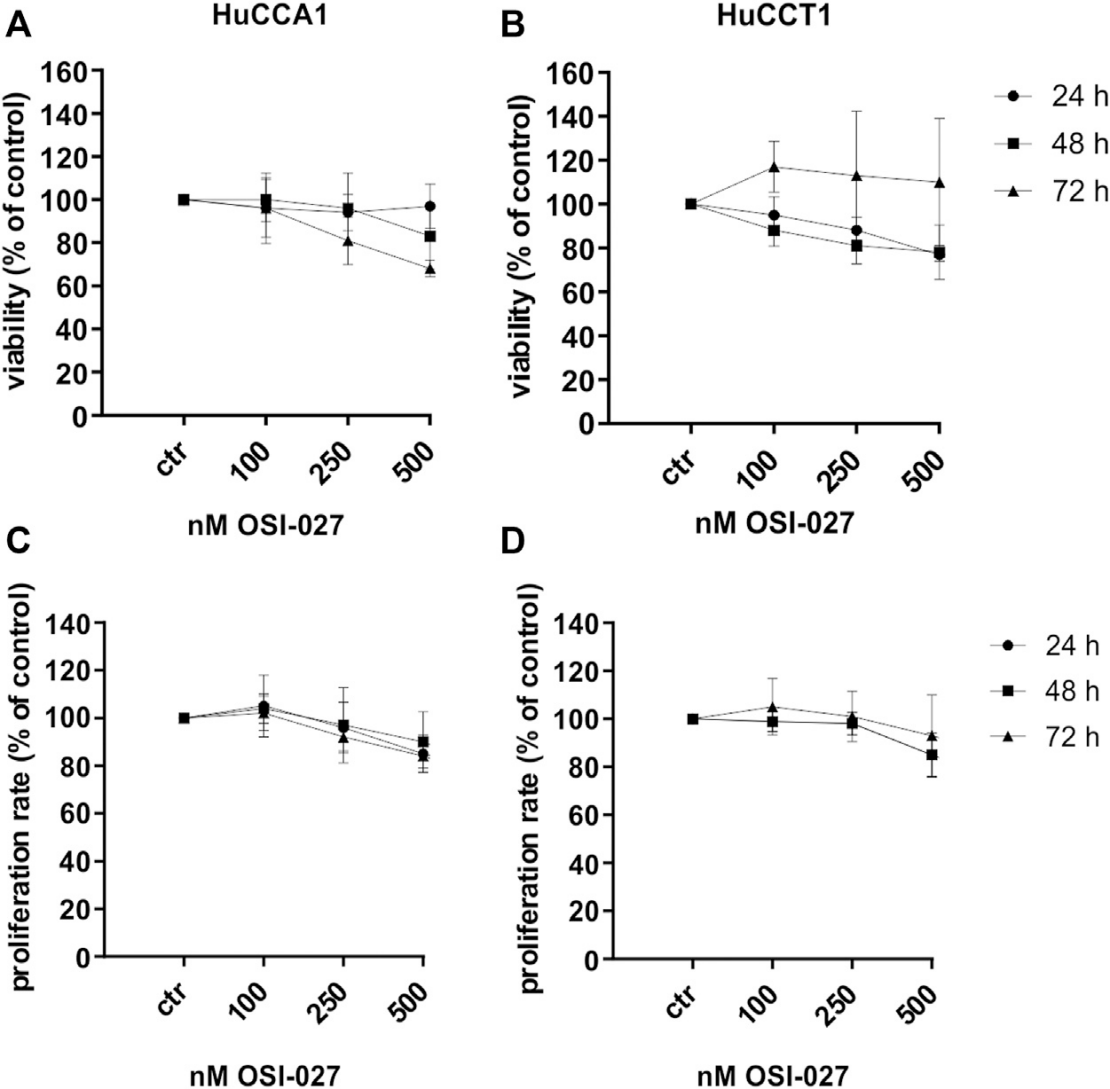
Dual inhibition of mTORC1/2 does not decrease cell viability and proliferation rate. Cytotoxic effect of up to 500 nM OSI-027 was assessed for HuCCA1 **(A)** and HuCCT1 **(B)** by MTT assay over 72 h. Viability was plotted relative to untreated controls set to 100% (±SD of three independent experiments). BrdU incorporation assay indicates no inhibition of proliferation rate in HuCCA1 **(C)** and HuCCT1 **(D)** over 72 h. Treatment was normalized to untreated controls (±SD of three independent experiments).

To exclude increased cell death as an explanation for the inhibition of the migration capacity cell viability was determined by MTT assay (Figures 3A,B) over 72 h with concentrations of 100–500 nM OSI-027 as well as proliferation rate by BrdU incorporation assay (Figures 3C,D) and cell cycle analysis (Supplementary Figure S1). Less than 32% of HuCCA1 cells underwent cell death induced by mTORC1/2 inhibition after 72 h with the highest dose of 500 nM and less than 19% of the cells were affected by 250 nM (Figure 3A). For HuCCT1 cells, less than 22% of the cells were dead at 500 nM and less than 19% after exposure to 250 nM after 48 h, whereas after 72 h of exposure, cell viability recovered completely (Figure 3B). Likewise, the influence of OSI-027 on the proliferation rate was never more than 16% for both cell lines after exposure to 500 nM over 72 h (Figures 3C,D). Cell cycle analysis revealed no significant differences between controls and treated cells (Supplementary Figures S1A–B). In summary, inhibition of mTORC1/2 with up to 500 nM OSI-027 does affect neither cell viability nor the proliferation rate of CCA cell lines *in vitro*.

### Exposure to OSI-027 Leads to Impaired Activation of AKT and MAPK Signaling in Cholangiocarcinoma

**FIGURE 4:**
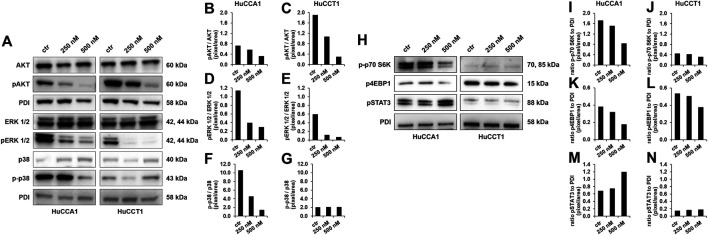
OSI-027 treatment reduces AKT and MAPK signaling. Representative western blot analysis of total AKT (60 kDa), pAKT^Ser473^ (60 kDa), total ERK 1/2 (42, 44 kDa), pERK^Thr202/Tyr204^ 1/2 (42, 44 kDa), p38 (40 kDa) and p-p38^Thr180/Tyr182^ (43 kDa) in human HuCCA1 and HuCCT1 after treatment with 250 and 500 nM OSI-027 as well as untreated (ctr) **(A)**. Analysis shows a dose-dependent reduction in anti-apoptotic AKT signaling in CCA cells as well as of ERK 1/2 and p38 survival and proliferation signaling. Densitometry of pAKT^Ser473^ phosphorylation is determined by ratio of pAKT^Ser473^/AKT **(B–C)**. Phosphorylation of pERK^Thr202/Tyr204^ 1/2 was determined by ratio of pERK^Thr202/Tyr204^ 1/2/ERK **(D–E)**. The activation of p38 as part of the MAPK signaling pathway was determined by ratio of p-p38^Thr180/Tyr182^/p38 **(F–G)**. OSI-027 treatment reduces the phosphorylation of the mTOR downstream effectors p70S6K^Thr389^ (70, 85 kDa) and 4EBP1^Ser65^ (15 kDa) in HuCCA1 and HuCCT1 after treatment with 250 and 500 nM as well as untreated control **(H)**. Survival signaling by phosphorylation of STAT3^Tyr705^ (88 kDa) is increased by dual kinase inhibition in HuCCA1 **(H)**. Densitometry of p-p70S6K^Thr389^, p4EBP1^Ser65^ and pSTAT3 ^Tyr705^ is determined by ratio to loading control **(I–N)**. 20 µg of protein lysates were separated by SDS-PAGE in three independent experiments, displayed are cropped blots. PDI was used as a loading control.

Protein analysis was used to determine the effect of OSI-027 on the activation of important targets of mTOR (Figure 4A). One of the main targets of mTOR inhibitors is the phosphorylation of the anti-apoptotic AKT at serine 473. As the results of the quantification shown, the activation of AKT was highly impaired up to 84% compared to untreated control in relation to total AKT in both the cell lines (Figures 4B,C). According to the dual inhibition of mTOR, the downstream effector molecules p-p70S6K and 4EBP1 were decreased by OSI-027 in HuCCA1 more obvious than in HuCCT1 (Figures 4H, I–L), while survival signaling by activation of STAT3 was slightly increased by dual kinase inhibition with OSI-027 in HuCCA1 (Figures 4H, M). The MAPK pathway was interrupted by impaired ERK and p38 activation after exposure to OSI-027. Cells exposed to 500 nM OSI-027 revealed a decrease of ERK-phosphorylation at threonine 202 and tyrosine 204 up to 83% in relation to total ERK (Figures 4D,E). The activation of MAPK p38 was reduced up to 87% for HuCCA1 (Figure 4F) in relation to total p38 but phosphorylation at threonine 180 and tyrosine 182 was not affected in HuCCT1 (Figure 4G). These results demonstrate that treatment with the dual-kinase inhibitor OSI-027 impairs important oncogenic signaling pathways, involved in cancer cell motility in CCA cell lines.

### OSI-027 Regulates the Protein Level of Matrix Metalloproteinases 2 and 9 in Cholangiocarcinoma

**FIGURE 5:**
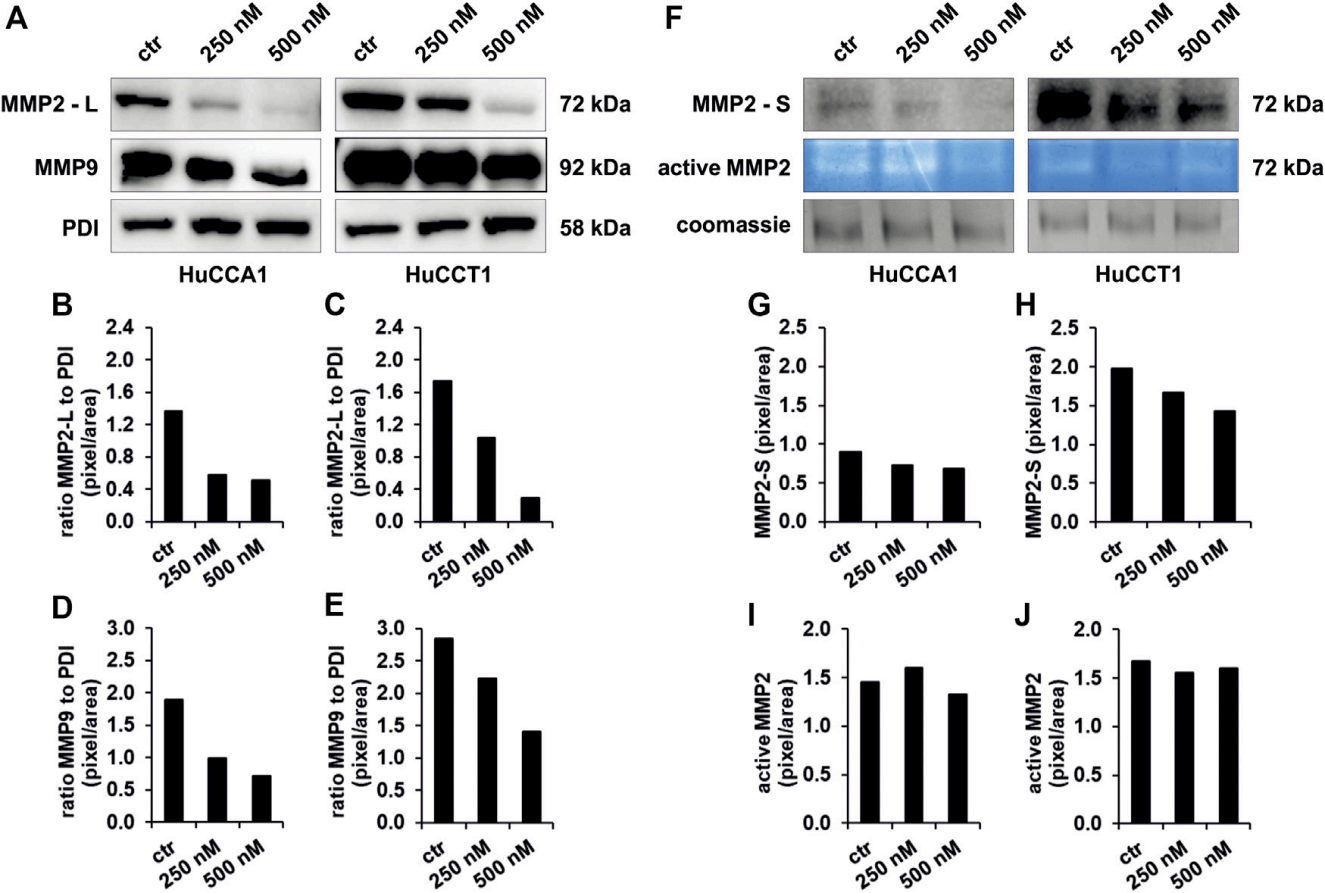
Exposure to OSI-027 reduces migration capacity by decrease of MMP2 and MMP9. Representative images **(A, F)** of western blot analysis and zymography analysis with conditioned media of HuCCA1 and HuCCT1 indicate less accumulation of MMP2 (-L) and MMP9 in the cell lysate as well as a decrease of secretion (MMP2-S) and few less activation of MMP2 in CCA cells when treated with 250–500 nM OSI-027.4 µg of protein of the supernatant and 20 µg of the cell lysates were screened for MMP2 and MMP9. PDI was used as loading control for cell lysates. Densitometry **(B–E and G–J)** reveals a dose-dependent decrease of MMP2 expression in the cells by up to 83% **(B and C)**. Expression levels of MMP9 are decreased by up to 63% **(D and E)**. The secretion of MMP2 in the supernatant is decreased as well by up to 25% **(G and H)**. The activity of the secreted MMP2 is decreased by 7% in HuCCA1 **(I)** but not constantly in HuCCT1 **(J)**.

MMPs are involved in tumor cell migration by degrading the extracellular matrix of the tumor tissue. In the present study, we found a dose-dependent decrease of protein expression of MMP2 in the lysate (MMP2—L) of CCA cells up to 83% after exposure to 250 and 500 nM OSI-027 for both the cell lines (Figures 5A–C). In addition, the expression level of MMP9 revealed a dose-dependent decrease in HuCCA1 up to 63% (Figure 5D) but no impaired expression in HuCCT1 (Figure 5E). Western blot of the conditioned media obtained from HuCCA1 and HuCCT1 cells showed a dose-dependent reduced protein level of MMP2 (MMP2—S) up to 25% with increasing concentrations of OSI-027 up to 500 nM (Figures 5F–H). Likewise, zymography analysis of the conditioned media in non-degrading gelatin gels revealed a reduced activation of MMP2 in HuCCA1 and HuCCT1 exposed to OSI-027 (Figures 5F–J). Taken together, dual-kinase inhibition of mTORC1/2 impairs the expression level and activation of MMP2 in CCA cells *in vitro*.

## Discussion

In this study, we assessed the role of mTORC1/2 inhibition in tumor cell survival and migration in iCCA. Using the dual-kinase inhibitor OSI-027, we found decreased migration capacity and surviving fraction but also maintained viability and proliferation. However, survival as well as anti-apoptotic signaling was reduced in treated iCCA cells. Finally, inhibition of mTORC1/2 signaling with OSI-027 impaired the expression of MMP2, MMP9, and the activation of MMP2 in iCCA cells.

The protein complex mTORC1 is known to be activated by PI3K/AKT and MAPK signaling pathways (Hay and Sonenberg 2004). These oncogenic pathways are frequently mutated, resulting in hyperactivation of mTORC1, which is found in many human cancers and associated with increased tumorigenesis (Hay 2005; Sarbassov et al., 2005; Rosen and She 2006). While rapalogs did not show the anticipated success in tumor therapy due to compensatory upregulation of AKT—the main downstream effector of mTORC2 signaling and one of the most frequently activated proteins in cancer - mTORC2/RICTOR gained more and more importance in cancer research. The upregulation of RICTOR was described in different kinds of cancerto be associated with an impaired prognosis (Bian et al., 2015; Schmidt et al., 2017; Wang et al., 2017). Moreover, RICTOR was shown to be involved in tumorigenesis and metastasis. Accordingly, we recently found that selective pharmacological inhibition of mTORC2 significantly decreased migration and invasion *in vitro* and the establishment of liver metastasis of melanoma cells *in vivo* (Guenzle et al., 2020). Moreover, the specific knockdown of RAPTOR and RICTOR caused a decrease in cell migration, suggesting their essential role in prostate cancer cell movement (Venugopal et al., 2020). In addition, RICTOR knockdown reduced cellular chemotactic capacity and ablates pulmonary metastasis in breast cancer (Zhang et al., 2010). Finally, we recently demonstrated a significant reduction of cell motility and liver metastasis by RNAi-mediated suppression of RICTOR in a melanoma mouse model (Schmidt et al., 2018) and a significant decrease of cancer cell motility when targeting mTORC2/RICTOR in addition to rapamycin treatment in gastric cancer cells (Lang et al., 2010). These findings emphasize that mTORC1 and mTORC2 are of particular importance for metastasis in various cancer entities and targeting both complexes might be a potential option for anti-neoplastic therapy.

Results from our experiments demonstrate that targeting mTORC1/2 using a dual kinase inhibitor significantly reduced migration and invasion capacity, one of the key steps in metastasis, in iCCA cells.

OSI-027 reduced the migration capacity in a dose and time-dependent manner. While migration capacity was rather delayed at early time points statistically different results between treated and control cells were reached after 48 h or 96 h of treatment. However, this might reflect the drug’s biologic dose-effect curve and therefore be translated into physiological consequences for tumor progression. Our findings are consistent with different studies showing an important role for mTORC1 as for mTORC2 in the migratory potential of cancer cells. When inhibiting mTORC1 and mTORC2, (Gulhati et al., 2011) found a significantly reduced migration of colorectal cancer cells by regulation of the GTPases RhoA and Rac1. Moreover, it was shown that mTORC1-mediated S6K1 and 4E-BP1 pathways are involved in the regulation of cell motility (Berven et al., 2004; Liu et al., 2006) and that rapamycin treatment blocked the cytoskeletal actin polymerization, a functional indicator of cell migration (Berven et al., 2004). When examining the role of mTORC2 in cancer cell migration, the protein complex was found to phosphorylate filamin A, an actin cross-linking protein to maintain focal adhesion dynamics and cell migration (Sato et al., 2016). Accordingly, knockdown of its central subunit RICTOR resulted in defective F-actin fiber formation and interruption of the cytoskeleton (Jacinto et al., 2004). Masri *et al.* showed that overexpression of RICTOR resulted in enhanced activity of PKCα, and promoted glioma cell proliferation, migration, and invasiveness (Masri et al., 2007). These data indicate that both mTORC1 and mTORC2 are involved in cancer cell migration by organization and regulation of the cytoskeleton. The arrangement of the cytoskeleton also functions as an important morphologic and biochemical pattern of the epithelial to mesenchymal transition (EMT), another crucial step of the metastatic cascade, in which mTORC1/2 is involved. Upon mTORC1/2 inhibition, increased cell-cell contacts, increased E-cadherin, decreased vimentin, and formation of lamellipodia, as well as decreased MMP9, were noticed which are all known as characteristic patterns of a reversed EMT, also known as mesenchymal to epithelial transition (MET) (Gulhati et al., 2011).

In this study, we also found mTORC1/2 inhibition to regulate MMPs. Particularly, MMP9 and MMP2 were reduced upon OSI-027 treatment. MMP9 is not only known to be associated with EMT but also with tumor cell migration and invasion. Zhou *et al.* revealed that cell migration and invasion are promoted due to an increased expression and proteolytic activity of MMP9, which plays an important role in the proteolytic destruction of the extracellular matrix and is, therefore, crucial for tumor invasion and metastasis (Zhou and Wong 2006). In line with these findings, a study showed that the activated PI3K-AKT-mTOR signaling pathway promotes invasion and metastasis in hepatocellular carcinoma through up-regulation of MMP9 (Chen et al., 2009). In breast cancer, MMP2 and MMP9 were determined as important downstream effectors of PI3K/AKT signaling (Zhou et al., 2014) and MMP2 was shown to be activated by PI3K/AKT, regulated by RICTOR for the vasculogenic mimicry of tumor cells (Liang et al., 2017). Here, we provide further evidence for a close connection between mTOR signaling, PI3K/AKT signaling, and activation of MMPs.

Similarly, MAPK signaling is also connected to MMPs. demonstrated that impaired activation of ERK1/2 and p38 by mTOR inhibition is directly involved in decreased MMP2 and MMP9 expression and, therefore, reduced migration and invasion in hepatocellular carcinoma (Lin et al., 2014). We also noticed the MAPK pathway to be interrupted by impaired ERK and p38 activation upon mTORC1/2 inhibition. This goes along with the findings of Reddy *et al.* who described ERK1/2 to be involved in the process of cell motility and invasion by mTORC1 mediated regulation of PP2A (Reddy et al., 2003; Harwood et al., 2008). These results suggest that rapalogs may inhibit cell motility by targeting the PP2A-ERK1/2 pathway.

In our study, the viability and the proliferation are not affected by the dual kinase inhibition. This is the particular importance since survival signaling is impaired in both the cell lines and even more anti-apoptotic and MAPK signaling is found to be decreased One might speculate that activation of alternative pathways might regulate and stabilize the viability of the cells. In fact, we found an upregulation of phosphorylated STAT3 which at least in part might explain our observation (Ma and Blenis 2009; Foster and Fingar 2010; Shull et al., 2015; Qin et al., 2016). Nonetheless, the lack of inhibitory effects on proliferation and viability may also provide the basis for the combination of OSI-027 with chemotherapeutic agents such as gemcitabine or cisplatin. However, further research is warranted to clarify this.

Studies investigating dual kinase inhibition in CCA are very limited. However, the findings of this study go along with the recently published results of Zhang *et al.* (Zhang et al., 2017) who showed effective mTORC1 and mTORC2 signaling suppression by MLN0128, another ATP-competitive mTOR kinase inhibitor, and inhibited cell growth of iCCA cell lines due to induction of apoptosis without affecting cell proliferation. When applying MLN0128 *in vivo*, mTORC1/2 inhibition led to disease stabilization in an early stage and partial tumor regression in a late stage of AKT/YAP127A induced iCCA in mice. Adding palbociclib, a CDK4/6 inhibitor, to MLN0128, could even potentiate its anti-tumor effects for iCCA (Song et al., 2019) making dual kinase inhibition of mTORC1/2 a promising treatment strategy for CCA. Dual kinase inhibition in CCA was also examined by Ewald *et al.* (Ewald et al., 2014) who showed a reduced proliferation rate of three CCA cell lines *in vitro* upon treatment with AZD8055. This effect was even stronger for combined treatment with AKT inhibitor MK-2206 and AZD8055. While studies examining the effect of ATP-competitive inhibitors for CCA are rare, encouraging data for example in hepatocellular carcinoma, laryngeal carcinoma or lung carcinoma led to the establishment of the first early clinical trials. However, they showed mixed results, especially due to high toxicity profiles requiring dose reductions which were below biological effects (Magaway et al., 2019). Nevertheless, patients with RICTOR amplification in gastric and small cell lung cancer were sensible to treatment with mTOR kinase inhibitors and were subsequently identified as a subgroup that responded to the treatment (Kim et al., 2017; Sakre et al., 2017).

In summary, our current study shows that inhibition of mTORC1 and mTORC2, using the dual kinase inhibitor OSI-027, diminishes oncogenic signaling in iCCA cells. Furthermore, treatment with the inhibitor leads to reduction of tumor cell motility and impairs expression of MMPs. Therefore, targeting mTORC1/2 might be an option for future therapy concepts in iCCA.

## Data Availability

The original contributions presented in the study are included in the article/Supplementary Material, further inquiries can be directed to the corresponding author.

## Supplementary Material

Supplementary Figure S1 HuCCA1 **(A)** and HuCCT1 **(B)** were treated with 100—500 nM OSI-027 and stained with DAPI showing DNA content distribution in histograms. G0-G1 and G2-M phase peaks are separated by S phase. Populations of phases are displayed in percent. Violet laser 405 nm.

## Abbreviations

4E-BP1, Eukaryotic translation initiation factor 4E-binding protein 1; AKT, Protein kinase B; ATP, Adenosine triphosphate; BrdU, Bromdesoxyuridin; CCA, Cholangiocellular carcinoma; iCCA, Intrahepatic cholangiocellular carcinoma; CDK4/6, Cyclin dependent kinase 4/6; ECM, Extracellular matrix; EMT, Epithelial–mesenchymal transition; ERK, Extracellular-signal regulated kinases; MAPK, Mitogen-activated protein kinase; MTT, 3-(4,5-Dimethylthiazol-2-yl)-2,5-diphenyltetrazoliumbromid; mTOR, Mammalian target of rapamycin; mTORC1, Mammalian target of rapamycin complex 1; mTORC2, Mammalian target of rapamycin complex 2; MMP2, Matrix metalloproteinase 2; MMP9, Matrix metalloproteinase 9; OSI-027, Trans-4-[4-Amino-5-(7-methoxy-1H-indol-2-yl)imidazo[5,1-f][1,2,4]triazin-7-yl]cyclohexanecarboxylic acid; PE, Plating efficiency; PI3K, Phosphoinositide 3-kinase; PP2A, Protein phosphatase 2; RAPTOR, Regulatory associated protein of mTOR; RICTOR, Rapamycin-insensitive companion of mTOR; RNAi, RNA interference; S6K, Ribosomal protein S6 kinase; Ser, Serine; SF, Surviving fraction; SGK1, Serum/glucocorticoid regulated kinase 1; Thr, Threonine; Tyr, Tyrosine.

## References

[B1] Barrandon Y. Green H. (1987). Three Clonal Types of Keratinocyte with Different Capacities for Multiplication. Proc. Natl. Acad. Sci. 84, 2302–2306. 10.1073/pnas.84.8.2302 2436229 PMC304638

[B2] Beaver C. M. Ahmed A. Masters J. R. (2014). Clonogenicity: Holoclones and Meroclones Contain Stem Cells. PLoS One 9 (2), e89834. 10.1371/journal.pone.0089834 24587067 PMC3935944

[B3] Bendell J. C. Kelley R. K. Shih K. C. Grabowsky J. A. Bergsland E. Jones S. (2015). A Phase I Dose‐escalation Study to Assess Safety, Tolerability, Pharmacokinetics, and Preliminary Efficacy of the Dual mTORC1/mTORC2 Kinase Inhibitor CC‐223 in Patients with Advanced Solid Tumors or Multiple Myeloma. Cancer 121 (19), 3481–3490. 10.1002/cncr.29422 26177599 PMC4832308

[B4] Berven L. A. Willard F. S. Crouch M. F. (2004). Role of the p70S6K Pathway in Regulating the Actin Cytoskeleton and Cell Migration. Exp. Cel Res. 296, 183–195. 10.1016/j.yexcr.2003.12.032 15149849

[B5] Bhagwat S. V. Gokhale P. C. Crew A. P. Cooke A. Yao Y. Mantis C. (2011). Preclinical Characterization of OSI-027, a Potent and Selective Inhibitor of MTORC1 and MTORC2: Distinct from Rapamycin. Mol. Cancer Ther. 10 (8), 1394–1406. 10.1158/1535-7163.MCT-10-1099 21673091

[B6] Bian Y. Wang Z. Xu J. Zhao W. Cao H. Zhang Z. (2015). Elevated Rictor Expression Is Associated with Tumor Progression and Poor Prognosis in Patients with Gastric Cancer. Biochem. Biophysical Res. Commun. 464 (2), 534–540. 10.1016/J.BBRC.2015.07.001 26159923

[B7] Brindley P. J. Bachini M. Ilyas S. I. Khan S. A. Loukas A. Sirica A. E. (2021). Cholangiocarcinoma. Nat. Rev. Dis. Primers 7 (1), 65. 10.1038/s41572-021-00300-2 34504109 PMC9246479

[B8] Chen J.-s. Wang Q. Fu X.-h. Huang X.-H. Chen X.-l. Cao L.-q. (2009). Involvement of PI3K/PTEN/AKT/MTOR Pathway in Invasion and Metastasis in Hepatocellular Carcinoma: Association with MMP-9. Hepatol. Res. 39 (2), 177–186. 10.1111/j.1872-034X.2008.00449.x 19208038

[B9] Ding X. Chaiteerakij R. Moser C. D. Shaleh H. Boakye J. Chen G. (2016). Antitumor Effect of the Novel Sphingosine Kinase 2 Inhibitor ABC294640 Is Enhanced by Inhibition of Autophagy and by Sorafenib in Human Cholangiocarcinoma Cells. Oncotarget 7 (15), 20080–20092. 10.18632/oncotarget.7914 26956050 PMC4991440

[B10] Ewald F. Nörz D. Grottke A. Hofmann B. T. Nashan B. Jücker M. (2014). Dual Inhibition of PI3K-AKT-MTOR- and RAF-MEK-ERK-Signaling Is Synergistic in Cholangiocarcinoma and Reverses Acquired Resistance to MEK-Inhibitors. Invest. New Drugs 32 (6), 1144–1154. 10.1007/s10637-014-0149-7 25152244

[B11] Foster K. G. Fingar D. C. (2010). Mammalian Target of Rapamycin (MTOR): Conducting the Cellular Signaling Symphony. J. Biol. Chem. 285 (19), 14071–14077. 10.1074/jbc.R109.094003 20231296 PMC2863215

[B12] Guenzle J. Akasaka H. Joechle K. Reichardt W. Venkatasamy A. Hoeppner J. (2020). Pharmacological Inhibition of MTORC2 Reduces Migration and Metastasis in Melanoma. Int. J. Mol. Sci. 22 (1), 30. 10.3390/ijms22010030 PMC7792954 33375117

[B13] Guenzle J. Garrelfs N. W. C. Goeldner J. M. Weyerbrock A. (2019). Cyclooxygenase (COX) Inhibition by Acetyl Salicylic Acid (ASA) Enhances Antitumor Effects of Nitric Oxide in Glioblastoma *In Vitro* . Mol. Neurobiol. 56, 6046–6055. 10.1007/s12035-019-1513-6 30715649

[B14] Guenzle J. Wolf L. J. Garrelfs N. W. C. Goeldner J. M. Osterberg N. Schindler C. R. (2017). ATF3 Reduces Migration Capacity by Regulation of Matrix Metalloproteinases via NFκB and STAT3 Inhibition in Glioblastoma. Cell Death Discov. 3, 17006. 10.1038/cddiscovery.2017.6 28250971 PMC5327503

[B15] Gulhati P. Bowen K. A. Liu J. Stevens P. D. Rychahou P. G. Chen M. (2011). MTORC1 and MTORC2 Regulate EMT, Motility, and Metastasis of Colorectal Cancer via RhoA and Rac1 Signaling Pathways. Cancer Res. 71 (9), 3246–3256. 10.1158/0008-5472.CAN-10-4058 21430067 PMC3085654

[B16] Harwood F. C. Shu L. Houghton P. J. (2008). MTORC1 Signaling Can Regulate Growth Factor Activation of P44/42 Mitogen-Activated Protein Kinases through Protein Phosphatase 2A. J. Biol. Chem. 283 (5), 2575–2585. 10.1074/JBC.M706173200 18056704

[B17] Hay N. Sonenberg N. (2004). Upstream and Downstream of MTOR. Genes Dev. 18 (16), 1926–1945. 10.1101/gad.1212704 15314020

[B18] Hay N. (2005). The Akt-MTOR Tango and its Relevance to Cancer. Cancer Cell 8 (3), 179–183. 10.1016/j.ccr.2005.08.008 16169463

[B19] Jacinto E. Loewith R. Schmidt A. Lin S. Rüegg M. A. Hall A. (2004). Mammalian TOR Complex 2 Controls the Actin Cytoskeleton and Is Rapamycin Insensitive. Nat. Cel Biol 6 (11), 1122–1128. 10.1038/ncb1183 15467718

[B20] Kim S. T. Kim S. Y. Klempner S. J. Yoon J. Kim N. Ahn S. (2017). Rapamycin-Insensitive Companion of MTOR (RICTOR) Amplification Defines a Subset of Advanced Gastric Cancer and Is Sensitive to AZD2014-Mediated MTORC1/2 Inhibition. Ann. Oncol. 28 (3), 547–554. 10.1093/ANNONC/MDW669 28028034

[B21] Lang S. A. Hackl C. Moser C. Fichtner-Feigl S. Koehl G. E. Schlitt H. J. (2010). Implication of RICTOR in the MTOR Inhibitor-Mediated Induction of Insulin-like Growth Factor-I Receptor (IGF-IR) and Human Epidermal Growth Factor Receptor-2 (Her2) Expression in Gastrointestinal Cancer Cells. Biochim. Biophys. Acta (Bba) - Mol. Cel Res. 1803 (4), 435–442. 10.1016/j.bbamcr.2010.01.009 20116405

[B22] Liang X. Sun R. Zhao X. Zhang Y. Gu Q. Dong X. (2017). Rictor Regulates the Vasculogenic Mimicry of Melanoma via the AKT-MMP-2/9 Pathway. J. Cel. Mol. Med. 21 (12), 3579–3591. 10.1111/jcmm.13268 PMC5706568 28699701

[B23] Lin J.-J. Su J.-H. Tsai C.-C. Chen Y.-J. Liao M.-H. Wu Y.-J. (2014). 11-Epi-Sinulariolide Acetate Reduces Cell Migration and Invasion of Human Hepatocellular Carcinoma by Reducing the Activation of ERK1/2, P38MAPK and FAK/PI3K/AKT/MTOR Signaling Pathways. Mar. Drugs 12 (9), 4783–4798. 10.3390/md12094783 25222667 PMC4178498

[B24] Liu L. Li F. Cardelli J. A. Martin K. A. Martin K. A. Blenis J. (2006). Rapamycin Inhibits Cell Motility by Suppression of MTOR-Mediated S6K1 and 4E-BP1 Pathways. Oncogene 25 (53), 7029–7040. 10.1038/sj.onc.1209691 16715128

[B25] Ma X. M. Blenis J. (2009). Molecular Mechanisms of MTOR-Mediated Translational Control. Nat. Rev. Mol. Cel Biol 10 (5), 307–318. 10.1038/nrm2672 19339977

[B26] Magaway C. Kim E. Jacinto E. (2019). Targeting MTOR and Metabolism in Cancer: Lessons and Innovations. Cells 8 (12), 1584. 10.3390/cells8121584 PMC6952948 31817676

[B27] Masri J. Bernath A. Martin J. Jo O. D. Vartanian R. Funk A. (2007). MTORC2 Activity Is Elevated in Gliomas and Promotes Growth and Cell Motility via Overexpression of Rictor. Cancer Res. 67 (24), 11712–11720. 10.1158/0008-5472.CAN-07-2223 18089801

[B28] Mateo J. Olmos D. Dumez H. Poondru S. Samberg N. L. Barr S. (2016). A First in Man, Dose-Finding Study of the MTORC1/MTORC2 Inhibitor OSI-027 in Patients with Advanced Solid Malignancies. Br. J. Cancer 114 (8), 889–896. 10.1038/bjc.2016.59 27002938 PMC4984800

[B29] O'Reilly K. E. Rojo F. She Q.-B. Solit D. Mills G. B. Smith D. (2006). MTOR Inhibition Induces Upstream Receptor Tyrosine Kinase Signaling and Activates Akt. Cancer Res. 66 (3), 1500–1508. 10.1158/0008-5472.CAN-05-2925 16452206 PMC3193604

[B30] Popova N. V. Jücker M. (2021). The Role of MTOR Signaling as a Therapeutic Target in Cancer. Int. J. Mol. Sci. 22 (4), 1743. 10.3390/ijms22041743 33572326 PMC7916160

[B31] Qin X. Jiang B. Zhang Y. (2016). 4E-BP1, a Multifactor Regulated Multifunctional Protein. Cell Cycle 15 (6), 781–786. 10.1080/15384101.2016.1151581 26901143 PMC4845917

[B32] Rattanasinganchan P. Leelawat K. Treepongkaruna S-a. Tocharoentanaphol C. Subwongcharoen S. Suthiphongchai T. (2006). Establishment and Characterization of a Cholangiocarcinoma Cell Line (RMCCA-1) from a Thai Patient. World. J. Gastroenterol. 12 (40), 6500–6506. 10.3748/wjg.v12.i40.6500 17072981 PMC4100638

[B33] Reddy K. B. Nabha S. M. Atanaskova N. (2003). Role of MAP Kinase in Tumor Progression and Invasion. Cancer Metastasis Rev. 22 (4), 395–403. 10.1023/A:1023781114568 12884914

[B34] Rosen N. She Q.-B. (2006). AKT and Cancer-Is it All mTOR? Cancer Cell 10 (4), 254–256. 10.1016/J.CCR.2006.10.001 17045203

[B35] Sakre N. Wildey G. Behtaj M. Kresak A. Yang M. Fu P. (2017). RICTOR Amplification Identifies a Subgroup in Small Cell Lung Cancer and Predicts Response to Drugs Targeting MTOR. Oncotarget 8 (4), 5992–6002. 10.18632/oncotarget.13362 27863413 PMC5351607

[B36] Sarbassov D. D. Guertin D. A. Ali S. M. Sabatini D. M. (2005). Phosphorylation and Regulation of Akt/PKB by the Rictor-MTOR Complex. Science 307 (5712), 1098–1101. 10.1126/science.1106148 15718470

[B37] Sato T. Ishii J. Ota Y. Sasaki E. Shibagaki Y. Hattori S. (2016). Mammalian Target of Rapamycin (MTOR) Complex 2 Regulates Filamin A-dependent Focal Adhesion Dynamics and Cell Migration. Genes Cells 21 (6), 579–593. 10.1111/gtc.12366 27059097

[B38] Saxton R. A. Sabatini D. M. (2017). MTOR Signaling in Growth, Metabolism, and Disease. Cell 169 (2), 361–371. 10.1016/J.CELL.2017.03.035 28388417

[B39] Schenone S. Brullo C. Musumeci F. Radi M. Botta M. (2011). ATP-competitive Inhibitors of MTOR: An Update. Curr. Med. Chem. 18 (20), 2995–3014. 10.2174/092986711796391651 21651476

[B40] Schmidt K. M. Dietrich P. Hackl C. Guenzle J. Bronsert P. Wagner C. (2018). Peter Dietrich, Christina Hackl, Jessica Guenzle, Peter Bronsert, Christine Wagner, Stefan Fichtner-Feigl, et alInhibition of MTORC2/RICTOR Impairs Melanoma Hepatic Metastasis. Neoplasia 20 (12), 1198–1208. 10.1016/j.neo.2018.10.001 30404068 PMC6224335

[B41] Schmidt K. M. Hellerbrand C. Ruemmele P. Michalski C. W. Kong B. Kroemer A. (2017). Claus Hellerbrand, Petra Ruemmele, Christoph W. Michalski, Bo Kong, Alexander Kroemer, Christina Hackl, Hans J. Schlitt, Edward K. Geissler, and Sven A. LangInhibition of MTORC2 Component RICTOR Impairs Tumor Growth in Pancreatic Cancer Models. Oncotarget 8 (15), 24491–24505. 10.18632/oncotarget.15524 28445935 PMC5421865

[B42] Shull A. Y. Noonepalle S. K. AwanAwan F. T. Liu J. Pei L. Bollag R. J. (2015). RPPA-based Protein Profiling Reveals EIF4G Overexpression and 4E-BP1 Serine 65 Phosphorylation as Molecular Events that Correspond with a Pro-survival Phenotype in Chronic Lymphocytic Leukemia. Oncotarget 6 (16), 14632–14645. 10.18632/oncotarget.4104 25999352 PMC4546493

[B43] Song X. Liu X. Wang H. Wang J. Qiao Y. Cigliano A. (2019). Combined CDK4/6 and Pan-MTOR Inhibition Is Synergistic against Intrahepatic Cholangiocarcinoma. Clin. Cancer Res. 25 (1), 403–413. 10.1158/1078-0432.CCR-18-0284 30084835 PMC6423983

[B44] Statz C. M. Patterson S. E. Mockus S. M. (2017). MTOR Inhibitors in Castration-Resistant Prostate Cancer: A Systematic Review. Targ Oncol. 12 (1), 47–59. 10.1007/s11523-016-0453-6 27503005

[B45] Venugopal S. V. Caggia S. Gambrell‐Sanders D. Khan S. A. (2020). Differential Roles and Activation of Mammalian Target of Rapamycin Complexes 1 and 2 during Cell Migration in Prostate Cancer Cells. Prostate 80 (5), 412–423. 10.1002/pros.23956 31995655 PMC7232714

[B46] Wang L. Qi J. Yu J. Chen H. Zou Z. Lin X. (2017). Overexpression of Rictor Protein in Colorectal Cancer Is Correlated with Tumor Progression and Prognosis. Oncol. Lett. 14 (5), 6198–6202. 10.3892/ol.2017.6936 29113267 PMC5661410

[B47] Zhang F. Zhang X. Li M. Chen P. Zhang B. Guo H. (2010). mTOR Complex Component Rictor Interacts with PKCζ and Regulates Cancer Cell Metastasis. Cancer Res. 70 (22), 9360–9370. 10.1158/0008-5472.CAN-10-0207 20978191

[B48] Zhang S. Song X. Cao D. Xu Z. Fan B. Che L. (2017). Pan-MTOR Inhibitor MLN0128 Is Effective against Intrahepatic Cholangiocarcinoma in Mice. J. Hepatol. 67 (6), 1194–1203. 10.1016/J.JHEP.2017.07.006 28733220 PMC5696057

[B49] Zhou H. Y. Wong S. T. (2006). Activation of p70S6KInduces Expression of Matrix Metalloproteinase 9 Associated with Hepatocyte Growth Factor-Mediated Invasion in Human Ovarian Cancer Cells. Endocrinology 147 (5), 2557–2566. 10.1210/en.2005-1404 16469801

[B50] Zhou R. Xu L. Ye M. Liao M. Du H. Chen H. (2014). Formononetin Inhibits Migration and Invasion of MDA-MB-231 and 4T1 Breast Cancer Cells by Suppressing MMP-2 and MMP-9 through PI3K/AKT Signaling Pathways. Horm. Metab. Res. 46 (11), 753–760. 10.1055/s-0034-1376977 24977660

