# Supplementary figures and images for: Dual Inhibition of mTORC1/2 Reduces Migration of Cholangiocarcinoma Cells by Regulation of Matrixmetalloproteinases
Source: Front Cell Dev Biol. 2022 Jan 13;9:785979. doi: 10.3389/fcell.2021.785979 (PMC8793831; doi:10.3389/fcell.2021.785979)

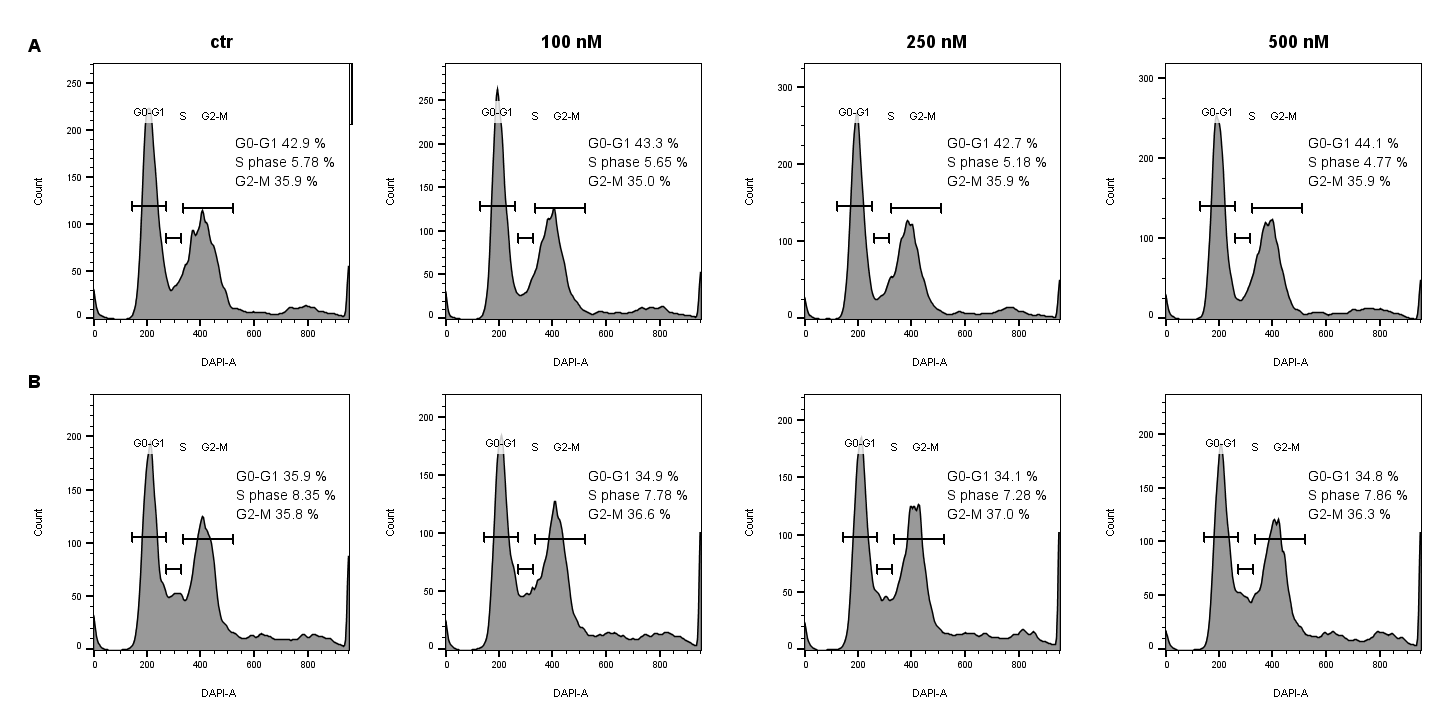

Supplement: Supplementary file 1 [file Image1.tiff]
